# Thalamic Shape Abnormalities Differentially Relate to Cognitive Performance in Early-Onset and Adult-Onset Schizophrenia

**DOI:** 10.3389/fpsyt.2022.803234

**Published:** 2022-04-11

**Authors:** Derin Cobia, Chaz Rich, Matthew J. Smith, Pedro Engel Gonzalez, Will Cronenwett, John G. Csernansky, Lei Wang

**Affiliations:** ^1^Department of Psychology and Neuroscience Center, Brigham Young University, Provo, UT, United States; ^2^Department of Psychiatry and Behavioral Sciences, Northwestern University Feinberg School of Medicine, Chicago, IL, United States; ^3^Department of Psychology, University of Notre Dame, Notre Dame, IN, United States; ^4^School of Social Work, University of Michigan, Ann Arbor, MI, United States; ^5^Department of Psychiatry and Behavioral Health, The Ohio State University Wexner Medical Center, Columbus, OH, United States

**Keywords:** psychosis, development, MRI, surface-mapping, neuroimaging

## Abstract

Early-onset schizophrenia (EOS) shares many biological and clinical features with adult-onset schizophrenia (AOS), but may represent a unique subgroup with greater susceptibility for disease onset and worsened symptomatology and progression, which could potentially derive from exaggerated neurodevelopmental abnormalities. Neurobiological explanations of schizophrenia have emphasized the involvement of deep-brain structures, particularly alterations of the thalamus, which have been linked to core features of the disorder. The aim of this study was to compare thalamic shape abnormalities between EOS and AOS subjects and determine whether unique behavioral profiles related to these differences. It was hypothesized abnormal thalamic shape would be observed in anterior, mediodorsal and pulvinar regions in both schizophrenia groups relative to control subjects, but exacerbated in EOS. Magnetic resonance T1-weighted images were collected from adult individuals with EOS (*n* = 28), AOS (*n* = 33), and healthy control subjects (*n* = 60), as well as collection of clinical and cognitive measures. Large deformation high-dimensional brain mapping was used to obtain three-dimensional surfaces of the thalamus. General linear models were used to compare groups on surface shape features, and Pearson correlations were used to examine relationships between thalamic shape and behavioral measures. Results revealed both EOS and AOS groups demonstrated significant abnormal shape of anterior, lateral and pulvinar thalamic regions relative to CON (all *p* < 0.007). Relative to AOS, EOS exhibited exacerbated abnormalities in posterior lateral, mediodorsal and lateral geniculate thalamic regions (*p* = 0.003). Thalamic abnormalities related to worse episodic memory in EOS (*p* = 0.03) and worse working memory (*p* = 0.047) and executive functioning (*p* = 0003) in AOS. Overall, findings suggest thalamic abnormalities are a prominent feature in both early- and late-onset schizophrenia, but exaggerated in EOS and have different brain-behavior profiles for each. The persistence of these abnormalities in adult EOS patients suggests they may represent markers of disrupted neurodevelopment that uniquely relate to the clinical and cognitive aspects of the illness.

## Introduction

The onset of psychosis during childhood or early adolescence provides a unique research opportunity to explore the etiology of schizophrenia since children and adolescents with early-onset schizophrenia may represent a more homogenous subgroup associated with severe developmental deficits and greater familial susceptibility for the disorder (1). Additionally, given abnormal neural development is thought to contribute to the modulation of schizophrenia, early-onset schizophrenia offers a window to study the well-established neurodevelopmental hypothesis for schizophrenia (2). The anatomical pattern and the timing of the illness is still unclear, hence studies of early-onset schizophrenia could offer further insight into the pathophysiological process of the disease to precisely differentiate between normal brain development and the disease-associated pathological development.

Previously, it was unknown whether early-onset schizophrenia was an earlier extension of adult-onset schizophrenia or if it represented an independent pathophysiological process (3). Examination of early-onset schizophrenia has provided evidence for the continuity between early- and adult-onset groups since both types broadly share many of the same physiological and psychopathological features (4, 5). However, early-onset schizophrenia has been consistently associated with more severe premorbid psychopathology and cognitive impairment (2), which could potentially derive from an exaggeration of the neurodevelopmental abnormalities usually present in schizophrenia. For example, patients with early onset typically present with more severe premorbid language, motor, and social delays than patients with later onset in adolescence (4). Moreover, a study exploring cognitive differences between first-episode adolescents and first-episode adults with schizophrenia found that early-onset patients performed poorly in language and working memory tasks, as well as exhibited greater motor performance deficits compared with adult-onset (6).

Recent theories about the neurobiology of schizophrenia have emphasized the involvement of deep-brain structures, particularly the thalamus (7, 8). Due to the central role the thalamus plays in the coordination of information flow and cognition (9), dysfunction of this region is often implicated in many of the cardinal symptoms of schizophrenia, such as disorganized thought and executive dysfunction among others (10). Developmentally, the thalamus plays a pivotal role in the genesis of the cerebral cortex, with thalamic input being critical for appropriate functional differentiation of the cortex and intercommunicating regions (11), which is consistent with the neurodevelopmental hypothesis of schizophrenia (2). Neuroimaging studies of early-onset schizophrenia have demonstrated similar, and sometimes more pronounced, patterns of structural brain abnormalities with respect to schizophrenia in general (12–14). There is evidence thalamic volume is globally reduced in early-onset subjects (15, 16), with specific volume loss in mediodorsal and pulvinar regions (17).

Most studies to date have examined the features of early-onset schizophrenia in child and adolescent populations (18, 19), with few investigating them later in the course of the illness. Furthermore, there are no studies that have specifically investigated subtle morphological alterations of the thalamus available through shape analytic procedures in early-onset schizophrenia and compared against a matched adult-onset group. The aim of the current study was to utilize high-dimensional surface-mapping to characterize regional abnormalities of the thalamus in well-matched adult groups of early-onset and adult-onset schizophrenia, as well as matched control participants, to assess the persistence of theorized neurobiological exacerbations of altered neurodevelopment in early-onset schizophrenia. Based on previous work (20), it was hypothesized that abnormal shape would be observed in anterior, mediodorsal and pulvinar regions in the schizophrenia groups, but exaggerated in early-onset schizophrenia. Furthermore, it was hypothesized that early-onset-associated shape changes would demonstrate stronger relationships with cognition and psychopathology than those associated with adult-onset schizophrenia.

## Materials and Methods

### Sample

Participants included 28 individuals with early-onset schizophrenia (EOS), 33 individuals with adult-onset schizophrenia (AOS) and 60 healthy control (CON) participants all group-matched (using random selection) with respect to age, gender, and parental SES. Given schizophrenia is associated with progressive gray matter loss (21, 22), AOS and EOS subjects were also group-matched based on duration of illness. Complete recruitment methods have been described previously (23). The project was approved by the IRB at Washington University in St. Louis, and informed consent was obtained from each subject after a complete description of the study was given.

### Clinical Measures

Diagnosis of schizophrenia was determined by the consensus of a research psychiatrist and trained research clinicians using the Structured Clinician Interview for DSM-IV Axis I Disorders [SCID, (24)]. The criteria for coding age of illness onset were adapted from other longitudinal studies of EOS (2) where early-onset was defined as illness onset before 18 years of age, and AOS as onset by 18 years of age or older. Schizophrenia participants were asked to identify the age at which their acute psychotic symptoms first took place, which was provided using self-report during the SCID, as well as cross-referenced with medical records and a research evaluation by a psychiatrist. Duration of illness was computed as years difference between age of illness onset and current age.

The SCID was also used to identify lifetime diagnosis of a substance-use disorder for alcohol, cannabis, cocaine, stimulants, hallucinogens, sedatives, and opioids. Antipsychotic medication for schizophrenia participants was assessed through self-report, with first- and second-generation antipsychotic (FGA and SGA) treatments quantitatively measured based on type, dosage amount, duration of use, and the calculation of chlorpromazine equivalents using published guidelines (25). Nicotine use was estimated using a semi-structured interview adapted from Sullivan et al. (26), and alcohol use *via* the Lifetime Alcohol Consumption Assessment Procedure (27).

### Clinical and Cognitive Assessments

A battery of neuropsychological measures assessing key cognitive domains affected in schizophrenia was administered to all participants (28); raw scores were converted into standardized scores then selected measures were factored into three cognitive domains: working memory, episodic memory, and executive functioning. An index of crystallized intelligence was also derived to estimate the generalized cognitive deficit in psychosis. Some missing data was observed for cognitive variables, which included three CON, two EOS, and two AOS individuals, who were not included in the analyses. Three psychopathology clusters (positive, negative, and disorganized symptoms) were assessed and calculated using global ratings from the Scale for the Assessment of Positive Symptoms (29) and the Scale for the Assessment of Negative Symptoms (29). A full description of the specific measures used is reported in previous work (23).

### Image Acquisition

Details of the image acquisition, surface mapping and analysis of subjects can be found in previously published reports (20, 30). Briefly, magnetic resonance scans were collected with a standard head coil on a Siemens Magnetom 1.5T (Erlangen, Germany) scanner using a turbo-FLASH sequence (repetition time = 20 ms, echo time = 5.4 ms, flip angle = 30°, 180 slices, FOV = 256 mm, matrix = 356 × 256, time = 13.5 min) that acquired 1 mm^3^ isotropic whole-head images. Total brain volume was estimated using an atlas scaling factor (ASF), which is the reciprocal of the determinant of the alignment matrix to Talairach atlas space, and signifies the extent that the brain volume contracts or expands during alignment (31). No between-group differences were observed in the ASF (*F*_2,117_ = 1.7, *p* = 0.19) and thus, was not used as a covariate in statistical analyses.

### Surface Mapping

Thalamic surfaces were generated using Large-Deformation High-Dimensional Brain Mapping (HDBM-LD) procedures (32), an atlas-based approach that utilizes diffeomorphic transformations which aligns a template image to a target (i.e., subject) image and allows independent matching of individual surface points to maintain unique morphological features of each subject (33, 34). Validity and reliability for mapping the thalamus were established in previous reports (20, 30). Prior to diffeomorphic transformations, anatomic landmarks were placed by expert raters who were blinded to the group status of the scan being landmarked, detailed landmarking procedures can be found in previous publications (32, 33).

### Statistical Analyses

Demographic and clinical characteristics were calculated using chi-squared statistics and analysis of variance (ANOVA) models. Group differences in cognition and psychopathology were also evaluated using ANOVA models.

To examine thalamic volume, a repeated-measures ANOVA was used with hemisphere as the within-subjects effect and group as the between-subjects effect. For thalamic shape, deformation values along each surface were calculated as a contrast from the sample mean based on triangulated surface points for all subjects. Next, a principal components analysis (PCA) was used to reduce the high dimensionality of the surfaces, yielding an orthonormal set of eigenvectors that represented variation in the shape of the structures (33). The first 15 eigenvectors of the PCA accounted for more than 90% of total shape variance (across subjects and hemispheres) and used for subsequent statistical modeling. To evaluate thalamic shape differences across groups, a multivariate analysis of variance (MANOVA) model was utilized with shape variation (using all 15 eigenvectors scores averaged across hemispheres) as the dependent variable, and group status (EOS, AOS, and CON) as a fixed effect. If the overall MANOVA statistic was significant, follow-up MANOVA models were used to identify whether specific significant group contrasts existed (EOS vs. CON and AOS vs. CON). For the EOS vs. AOS contrast, a follow-up multivariate analysis of covariance (MANCOVA) model was used to account for the potentially confounding effects of illness duration, medication, and lifetime presence of a substance use disorder, which were included as covariates.

Visualization of group differences in thalamic shape deformation was accomplished by the construction of vertex-wise studentized-t contrast maps of the composite surfaces for each group. Shape displacements were calculated at each surface point as the difference between the means of the group vectors in magnitude and coded using a colored scale; final maps reflected corrected *p*-values using a familywise error rate approach based on random field theory with a vertex-wise threshold of *p* < 0.05 and a cluster-wise threshold of *p* < 0.01 (35). Inward and outward displacements, or deformations, of the surface were estimated as representations of localized volume loss or exaggeration at the neurobiological level (36).

To evaluate the relationship between thalamic shape and behavioral measures, a canonical score was first computed as a representation of composite shape based on all left-right averaged eigenvectors scores of the thalamus (37). Bivariate Pearson correlation coefficients were then calculated between the thalamic canonical shape score and measures of cognition (working memory, episodic memory, and executive functioning) and psychopathology (positive, negative, and disorganized symptoms) separately for EOS and AOS groups.

### Sensitivity Power Analysis

Sample sizes for the groups were fixed as data was derived from an archival schizophrenia dataset (8, 38). Sensitivity power analyses were calculated for the proposed models above to determine the smallest possible effect that could be detected from the data considering sample size restrictions. Using G*Power (39), it was determined that at 80% power, with a type I error rate of 0.05, and a combined sample of 121, there was power to detect the following minimal Cohen’s *f* effect sizes (40): ANOVA models for cognition = 0.29, and psychopathology = 0.26; thalamic volume RM-ANOVA = 0.28 (group effect). For the thalamic surface shape models, minimum Critical *F*-values were identified using G*Power for the main group effect given their multivariate nature: Three-group MANOVA = 1.51; 2-group MANOVAs for EOS vs. CON = 1.81, EOS vs. AOS = 1.89, AOS vs. CON = 1.79. For the correlation analyses, at a type I error rate of 0.05, there was 80% power to detect a correlation as large as: *r* = ±0.37 in the EOS group (*n* = 28); and *r* = ±0.34 in the AOS group (*n* = 33). The sample appears adequately powered to address the proposed research hypotheses, with the ability to, at a minimum, detect moderate effects (41). Cohen’s *f* values were calculated using criteria from Cohen (40) and Lenhard and Lenhard (42).

## Results

### Demographic, Clinical, and Confounding Variables

Anti-psychotic medication treatment has known effects on brain structure (43), while nicotine has been associated with reduced gray matter density (44), and a history of substance-use disorder can also affect brain morphometry (45). Given these findings, potential group differences for these confounds were examined; demographic and clinical variables are summarized in Table 1. Groups differed with respect to nicotine use, and lifetime histories of substance use disorders for alcohol, cannabis, cocaine, opiates, and sedatives. These variables were subsequently examined as fixed effect covariates in EOS vs. AOS linear models, with an aggregate measure (any lifetime history of a substance use disorder = 1, no lifetime history = 0) used for substance use. The EOS and AOS subjects also differed on mean dose years of first-generation antipsychotic treatment using the chlorpromazine equivalent, which was also included as a fixed effect covariate.

**TABLE 1:** Demographic and clinical characteristics of study sample.

	CON (*n* = 60)		EOS (*n* = 28)		AOS (*n* = 33)		Statistic		
	Mean	(SD)	Mean	(SD)	Mean	(SD)	*F*-test	df	*p*
Age, mean (SD)	36.2	(13.9)	34.7	(14.0)	40.1	(11.7)	1.40	2,118	0.25
Age of illness onset, mean (SD)	−	−	13.8	(3.0)	22.2	(4.1)	81.73	1,60	**<0.001**
Duration of illness, mean (SD)	−	−	21.0	(14.1)	17.9	(12.5)	0.82	1,60	0.37
Cigarette use (cigarettes per year)*	1,389	(2,892)	4,404	(4,699)	5,065	(5,525)	9.98	2,119	**<0.001**
Antipsychotic medication use									
1st-generation (Dose years)	−	−	0.3	(1.1)	3.0	(5.3)	7.36	1,60	**0.009**
2nd-generation (Dose years)	−	−	3.2	(2.9)	3.7	(3.6)	0.30	1,60	0.58
**Cognition**									
Crystallized IQ^†^	0.45	(0.87)	−0.39	(1.0)	−0.44	(0.77)	13.6	2,113	**<0.001**
Working memory^†^	0.38	(0.63)	−0.61	(0.67)	−0.42	(0.55)	29.8	2,113	**<0.001**
Episodic memory^†^	0.68	(0.66)	−0.56	(0.74)	−0.61	(0.64)	50.5	2,113	**<0.001**
Executive functioning^†^	0.41	(0.49)	−0.46	(0.76)	−0.57	(0.77)	32.5	2,113	**<0.001**
**Psychopathology**									
Positive symptoms	−	−	0.31	(0.82)	0.18	(0.84)	0.38	1,60	0.53
Negative symptoms	−	−	0.35	(0.66)	0.41	(0.64)	0.16	1,60	0.68
Disorganized symptoms	−	−	0.17	(0.67)	0.21	(0.56)	0.08	1,60	0.77

	***N***	**(%)**	***N***	**(%)**	***N***	**(%)**	**X ^2^**	**df**	***p***

Gender, No. (% male)	36	(60.0%)	17	(60.7%)	21	(63.6%)	0.12	2	0.94
SES class (Class 3)	25	(42.4%)	9	(40.95)	5	(17.2%)	13.56	8	0.94
**Substance use disorder**									
Alcohol	9	(15.0%)	13	(46.4%)	13	(39.4%)	11.59	2	**0.003**
Cannabis	2	(3.3%)	15	(53.6%)	7	(21.2%)	30.35	2	**<0.001**
Cocaine	1	(1.7%)	9	(32.1%)	2	(6.1%)	20.60	2	**<0.001**
Opiates	1	(1.7%)	4	(14.3%)	1	(3.0%)	6.81	2	**0.03**
Hallucinogens	1	(1.7%)	4	(14.8%)	1	(3.0%)	7.14	2	0.28
Sedatives	1	(1.7%)	5	(18.5%)	2	(6.1%)	8.52	2	**0.01**
Stimulants	1	(1.7%)	1	(3.7%)	2	(6.1%)	1.29	2	0.52

*EOS > CON (p = 0.002); AOS > CON (p < 0.001).

*^†^EOS < CON (p < 0.001); AOS < CON (p < 0.001). Bold = p < 0.05 for the overall omnibus model.*

### Cognition and Psychopathology

Cognition was compared across all three groups using ANOVA models with group as a fixed factor. Results (see Table 1) revealed a significant main effect of group for crystallized intelligence (*F*_2,113_ = 13.7, *p* < 0.001, Cohen’s *f* = −2.6), working memory (*F*_2,113_ = 29.8, *p* < 0.001, Cohen’s *f* = 0.65), episodic memory (*F*_2,113_ = 50.5, *p* < 0.001, Cohen’s *f* = 0.77), and executive functioning (*F*_2,113_ = 32.6, *p* < 0.001, Cohen’s *f* = 0.62). For all four cognitive domains, CON scored significantly higher than EOS and AOS (all *p*-values < 0.001). Contrasts between EOS and AOS did not achieve statistical significance (all *p*-values > 0.10). Results from ANOVA models evaluating EOS and AOS group differences on positive, negative, and disorganized symptoms were all non-significant.

### Thalamic Volume Analyses

For the volume of the thalamus, there was a significant main effect for hemisphere (*F*_1,118_ = 13.5, *p* < 0.001), but not for group (*F*_2,118_ = 2.93, *p* = 0.06, Cohen’s *f* = 0.173) or a group-by-hemisphere interaction (*F*_2,118_ = 0.83, *p* = 0.44).

### Thalamic Shape Analyses

A MANOVA model of thalamic eigenvectors (averaged across hemispheres) revealed an overall significant main effect of group on shape metrics (*F*_2,118_ = 2.4, *p* < 0.001). *Post hoc* comparisons using two-group MANOVA designs found significant differences between EOS (*F*_1,86_ = 2.4, *p* = 0.007) and AOS (*F*_1,91_ = 2.6, *p* = 0.003) versus CON. In the EOS vs. AOS MANCOVA, there was a significant main effect for group (*F*_15,40_ = 2.44, *p* = 0.012), as well as duration of illness (*F*_15,40_ = 4.3, *p* < 0.001), but not first-generation antipsychotic use, cigarette usage, or lifetime presence of a substance use disorder on the model. Notably, all of the significant *F*-values surpassed the Critical F thresholds calculated from the sensitivity analyses above.

**FIGURE 1:**
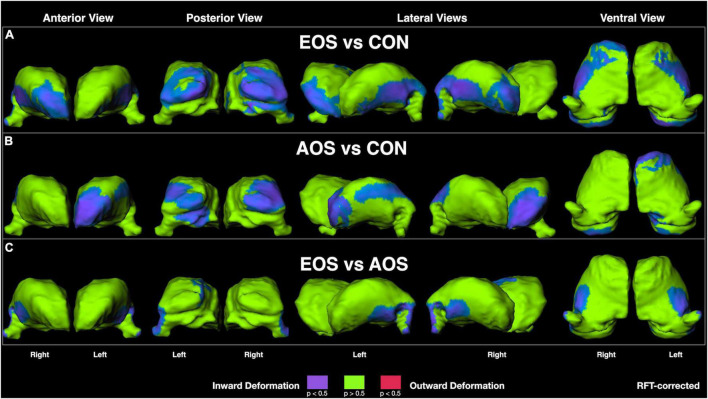
Thalamic surface shape displacement maps between: **(A)** Early-Onset Schizophrenia (EOS) patients and control (CON) participants; **(B)** Adult-Onset Schizophrenia (AOS) patients and CON participants; and **(C)** EOS and AOS patients. Cooler colors indicate significant regions of inward shape differences and warmer colors indicate significant regions of outward shape differences corrected for multiple comparisons using random field theory (RFT).

Visualization of RFT-corrected thalamic shape maps (Figure 1) revealed that EOS was characterized by prominent inward deformation, indicative of localized volume loss, in ventral lateral and lateral geniculate nuclei, as well as in anterior (right only) and pulvinar nuclei relative to CON (Figure 1A). For AOS, notable inward deformations were also observed in pulvinar nuclei in addition to left-sided anterior and lateral regions relative to CON (Figure 1B). Regarding the comparison between EOS and AOS, prominent inward deformations in EOS were observed in posterior ventral and left dorsal regions, as well as in the lateral geniculate nuclei (Figure 1C).

### Correlation Analyses

**FIGURE 2:**
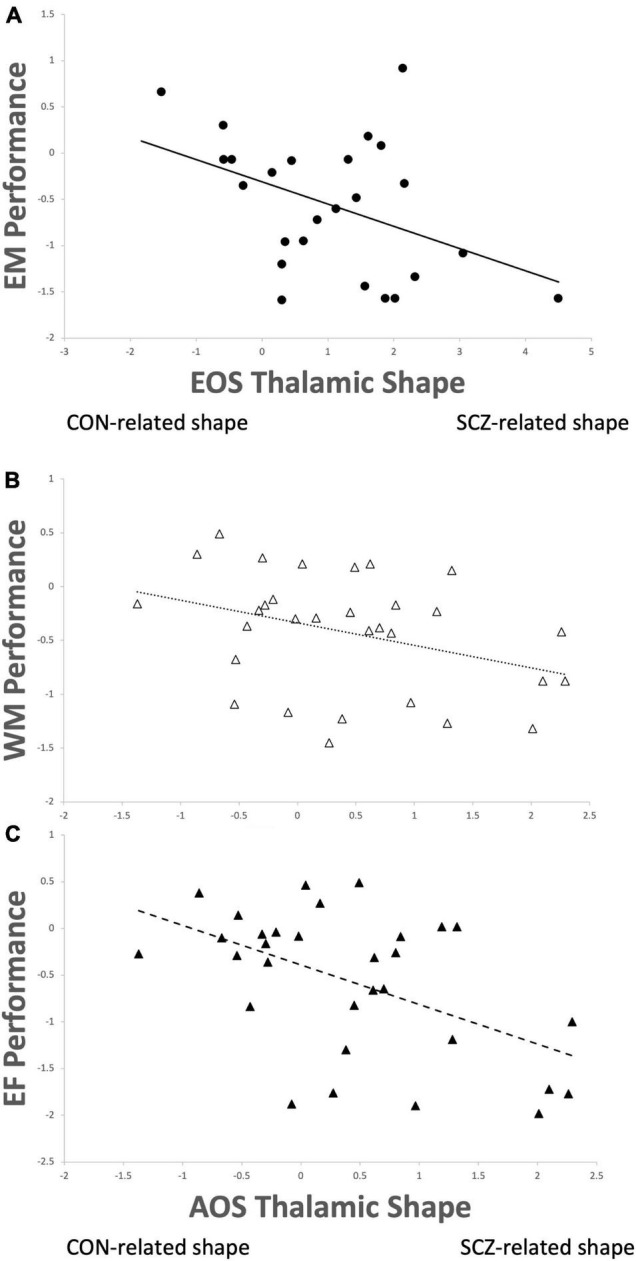
Thalamic shape progressing from healthy control (CON) to schizophrenia (SCZ) correlated with **(A)** poorer episodic memory performance (*r* = –0.43, *p* = 0.03) in early-onset schizophrenia (EOS); **(B)** poorer working memory performance (*r* = –0.36, *p* = 0.047) and **(C)** poorer executive functioning (*r* = –0.52, *p* = 0.003) in adult-onset schizophrenia (AOS).

Calculation of canonical scores for thalamic shape revealed increases in these values equated to greater shape abnormality (i.e., more disparate from the surface shape of the healthy comparison subjects). An outlier canonical score was observed in a single EOS participant (>3 SD above the mean), which was adjusted in to the 3 SD value using Winsorization procedures. In the EOS group, there was an inverse correlation between episodic memory scores and thalamic shape (*r* = −0.43, *p* = 0.03; Figure 2A), such that more abnormal thalamic surface shape related to poorer episodic memory performance. In the AOS group, a similar inverse correlation was observed between thalamic shape and working memory (*r* = −0.36, *p* = 0.047; Figure 2B) and executive functioning (*r* = −0.52, *p* = 0.003; Figure 2C). No other correlations between cognition or psychopathology and brain structure were significant (all *p*-values > 0.10).

## Discussion

Age of onset continues to represent an important factor for understanding and conceptualizing the pathology associated with the development of schizophrenia (46). This study sought to examine whether morphological differences of the thalamus, a highly implicated structure in the pathophysiology of psychosis, exist between early-onset and adult-onset schizophrenia in adulthood. Findings revealed a hemispheric difference in thalamic volume, but only modest differences between the schizophrenia and control groups, and no significant differences between EOS and AOS. However, shape analysis revealed significant thalamic abnormalities in EOS relative to CON in multiple anterior, posterior, and lateral regions; with similar patterns observed in AOS relative to CON. Examination of differences between the psychosis groups revealed exaggerated localized volume loss in EOS relative to AOS in ventral posterior and medial regions. Multivariate eigenvector models were also highly significant and support the observed vertex-wise group differences. Finally, unique relationships between shape and cognition were noted in the psychosis groups, with EOS demonstrating increased episodic memory impairment, and AOS worse working memory and executive functions, as thalamic shape became increasingly abnormal. Overall, these findings reveal the exaggerated effects of early-onset psychosis in adulthood on a brain structure critical to the pathophysiology of schizophrenia.

Early-onset schizophrenia is described as a condition with greater developmental and premorbid departures relative to AOS, likely due to a stronger genetic component to their presentation (2). This exacerbated presentation often leads to poorer clinical and cognitive outcomes (47), which has prompted considerations for focused treatments (48). Furthermore, unique brain abnormalities observed in EOS are generally more neurobiologically severe that in AOS (4). Summarized by Brent et al. (49), the most consistent findings include cortical abnormalities of frontal, temporal, and parietal regions, in addition to reduced global cerebral and cerebellar volumes. Furthermore, there is strong support for reduced thalamic volumes in EOS (50–52), with some evidence for progressive loss over time (17). While previous work has examined the gross anatomical volumetrics of the thalamus in EOS, the regional specificity of these abnormalities has yet to be characterized. This is especially relevant given the diverse connectivity matrix and unique nuclei specialization contained within the organization of the thalamus (9). Results from the current study revealed distinct patterns of abnormal shape, representative of localized volume loss, in EOS participants relative to AOS and healthy-matched individuals. Specifically, EOS was noted to have widespread inward deformations in ventral lateral regions, lateral geniculate nuclei, and in anterior and pulvinar nuclei relative to CON. The AOS group demonstrated a similar pattern relative to CON, but with more diffuse changes in lateral aspects and relative sparing in right regions. When the schizophrenia groups were compared directly against each other, it was observed EOS showed significant abnormal inward deformation in posterior ventral and dorsal regions, and in the lateral geniculate nucleus. The results suggest a pattern of abnormal thalamic shape in EOS that is similar to, but exacerbated, relative to AOS, which strongly implicates and supports a continuum model for the neurobiology of schizophrenia (53). Our finding is consistent with other neuroimaging work that found cortical gray matter loss in EOS is exaggerated, but mimics that in AOS (54). Furthermore, changes in global gray matter brain volume also support a neurodevelopmental continuum in psychosis as noted in a study examining these features in the offspring of probands with schizophrenia (55). Overall, neuroimaging markers, especially those involved in the pathophysiology of schizophrenia such as the thalamus, appear to be a robust approach for supporting a dimensional model of disease onset in psychosis-spectrum disorders (56).

Our pattern of findings within these onset types are broadly consistent with previous work on thalamic morphology from our group using different derivations of the sample, and include primarily alterations in anterior and posterior extremes in chronic cases (20), and a similar presentation in siblings (30). However, research using different methodology and sample compositions also provide consistent support for our results. For example, a study conducted by Janssen et al. (17) on thalamic volumes in an adolescent sample of male-only early-onset psychosis patients revealed regional volume loss in anterior mediodorsal and pulvinar areas in the right thalamus using a surface-based approach. In addition, using a combined voxel-based morphometry and novel thalamic nuclei segmentation procedure, Huang et al. (57) identified smaller pulvinar volumes in a large sample of youths with psychotic spectrum disorders. And in another recent report, Zhang et al. (58) observed abnormal functional connectivity (both hyper-and hypoconnectivity relative to healthy individuals) in the thalamocortical circuits of an EOS sample that included lateral and mediodorsal nuclei. While there is agreement between the above studies, the absence of anterior and ventral lateral abnormalities which was observed in our work is noted. This discrepancy could reflect the mean age difference in the samples used; as previously noted, our sample consisted of adult-aged EOS subjects while others were of early-onset adolescents. Thus, our findings may reflect an exaggerated pattern of abnormality that occurs as EOS ages into adulthood, which meaningfully informs an anticipated trajectory of development for these individuals, particularly in reference to AOS counterparts.

Cognitive dysfunction is a known feature in EOS (59) and has a general profile similar to that observed in AOS (60). We found that across various cognitive domains both EOS and AOS were significantly impaired relative to the healthy control group in crystallized IQ, working memory, episodic memory, and executive functioning, but did not significantly differ from each other in these domains. This is consistent with the known level of impairment observed in previous work on EOS where aspects of working memory, episodic memory and executive functioning are generally impaired to the same degree at AOS (60). The only exception to this literature is we found no difference between groups in crystallized IQ where other studies have found this domain to be more impaired in EOS (60). Again, it is important to note our comparisons were conducted on adult-aged patients, regardless of onset status, the EOS cognitive profiles we examined were ∼20 years post-onset. Work examining the longitudinal course of cognition in EOS as they transition into adulthood observes a broad attenuation of cognitive development relative to peers with no further decline after that (59, 61–63). Thus, it appears cognitive trajectories of EOS mimic that of AOS into adulthood.

Studies of neuroimaging markers for cognitive impairment in AOS are relatively plentiful (64–67), while much fewer have been conducted in EOS (68, 69). The behavioral substrates of affected thalamic nuclei we observed from the unique shape deformation patterns in EOS and AOS suggests these brain features may partially explain their observed cognitive impairment. In particular, higher-order aspects of cognitive control are known to involve the mediodorsal thalamus (70–72), while attentional and memory processes involve the pulvinar (73), and the anterior thalamus is implicated in episodic memory as part of Papez’ circuit (74). Clues regarding the relevance of thalamic involvement in cognition are well-detailed in experimental studies of animal mechanisms. For example, interrogations of mediodorsal nuclei in mice has revealed that excitation of this region is critical for sustaining task-related activity of the prefrontal cortex (75), and can also assist in modulating decision-making abnormalities *via* separate pathways between these areas (76). Linking disruption of thalamocortical circuitry with behavioral dysfunction in animal models such as these has yielded new insights into the pathophysiology of schizophrenia (77), and reinforced many findings from human imaging studies such as those described here. As such, abnormalities of the thalamus are increasingly considered putative biomarkers of schizophrenia given their endophenotypic potential with cognitive functioning and predictive ability for functional outcome and disease burden (78). While both EOS and AOS groups in our study demonstrated significant cognitive-thalamic relationships, the nature of these relationships differed. For EOS, it appears thalamic shape abnormalities in combined bilateral pulvinar and ventrolateral, with right anterior regions, strongly related to poorer performance in episodic memory. For AOS it was also bilateral pulvinar, but also left anterior and dorsolateral regions that related to poorer working memory and executive functioning. Given cognitive dysfunction in all these domains is a common feature for both groups, it is interesting to observe that a potential substrate for the impairment was *not* common. The development of brain features in EOS clearly differs from that of AOS, both in pattern and timing (18, 79–81). How these features relate to the maturation of cognitive functioning across childhood, adolescence and eventually adulthood is unclear and not well-studied. Our findings provide some insight into this process insomuch that, at least in adulthood, the possible underlying mechanisms of equivalently observed dysfunction are ultimately separate. This highlights the persistent conversation of heterogeneity in schizophrenia and its relevance to diagnosis, progression, treatment, and outcome (82–85).

One primary limitation to our study was the relatively small sample sizes of each group. To address this we conducted a sensitivity analysis for each model, which revealed sufficient power to detect even small-to-moderate effects in the aforementioned analyses. Given many of the Cohen’s *f* effect sizes, critical *F*, and *r* values for the shape MANOVA models and correlations were moderate-to-large, we believe the findings were not likely spurious or underpowered.

## Conclusion

Our research findings suggest that abnormalities of the thalamus are a prominent feature in both early- and late-onset schizophrenia. Using shape analyses we determined that the region pattern of these abnormalities was relatively similar between the early and late onset groups, occurring primarily in pulvinar, anterior and lateral regions. However, early-onset subjects demonstrated exaggerated abnormalities in ventral, left dorsomedial and lateral geniculate regions relative to adult-onset. Interestingly, abnormal thalamic shape features differentially related to cognition in each group – episodic memory for early-onset and working memory and executive functioning for adult-onset. These differences may be potentially useful as markers to understanding the developmental effects of schizophrenia onset on the neurobiology and cognitive functioning of this condition.

## Data Availability Statement

The datasets presented in this study can be found in online repositories. The names of the repository/repositories and accession number(s) can be found below: http://schizconnect.org.

## Ethics Statement

The studies involving human participants were reviewed and approved by the IRB at Washington University in St. Louis, St. Louis, MO, United States. The patients/participants provided their written informed consent to participate in this study.

## Author Contributions

DC and MS were responsible for study design, data analysis and interpretation, and drafting of the manuscript. CR and PE were responsible for data analysis and manuscript drafting. WC was responsible for manuscript drafting. JC and LW were responsible for study design and data analysis and interpretation. All authors contributed to the article and approved the submitted version.

## Conflict of Interest

JC is a consultant for Indivior Pharmaceuticals. DC is a consultant for Sage Pharmaceuticals. The remaining authors declare that the research was conducted in the absence of any commercial or financial relationships that could be construed as a potential conflict of interest.

## Publisher’s Note

All claims expressed in this article are solely those of the authors and do not necessarily represent those of their affiliated organizations, or those of the publisher, the editors and the reviewers. Any product that may be evaluated in this article, or claim that may be made by its manufacturer, is not guaranteed or endorsed by the publisher.
